# Supplementary material for: Investigating the Role of TGF-β Signaling Pathways in Human Corneal Endothelial Cell Primary Culture
Source: Cells. 2023 Jun 14;12(12):1624. doi: 10.3390/cells12121624 (PMC10297110; doi:10.3390/cells12121624)
Supplement: Supplementary file 1 [file cells-12-01624-s001.zip › cells-2380802-supplementary.pdf]

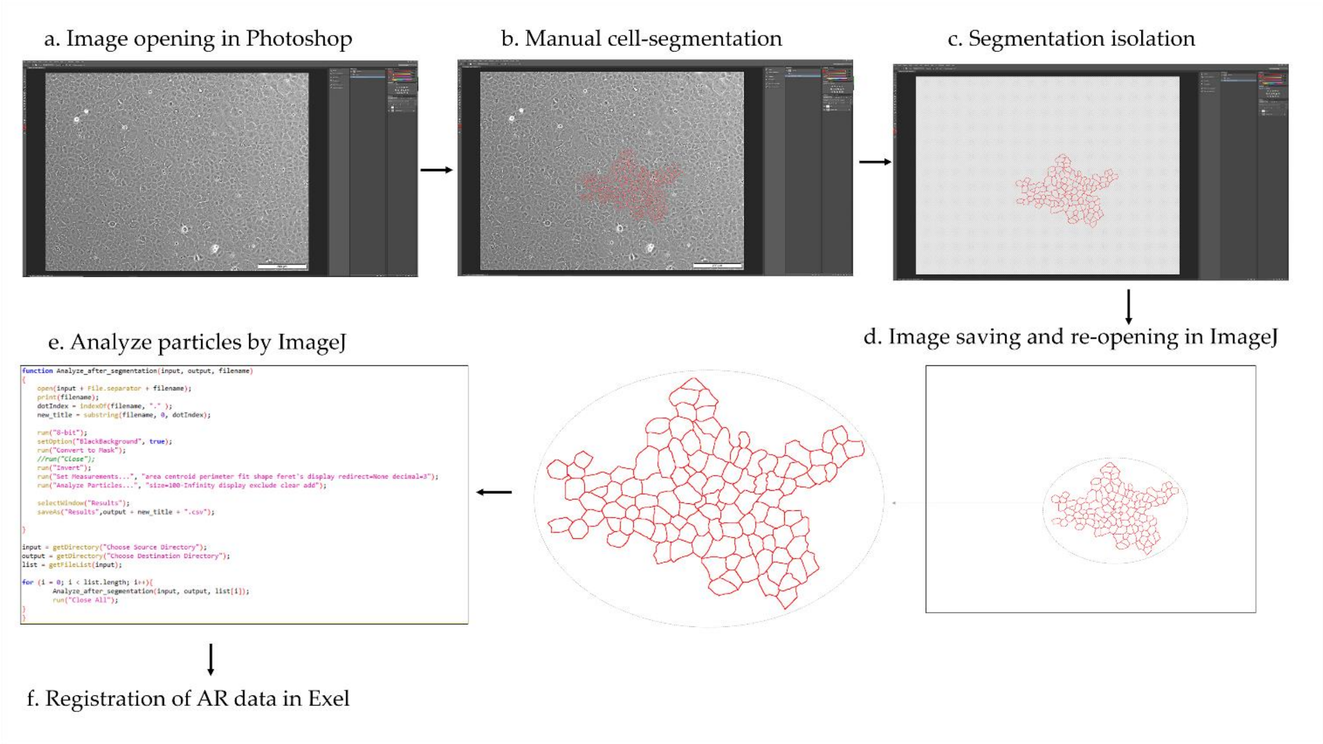

Supplementary Data S1. EndMT analysis process based on cell shape.

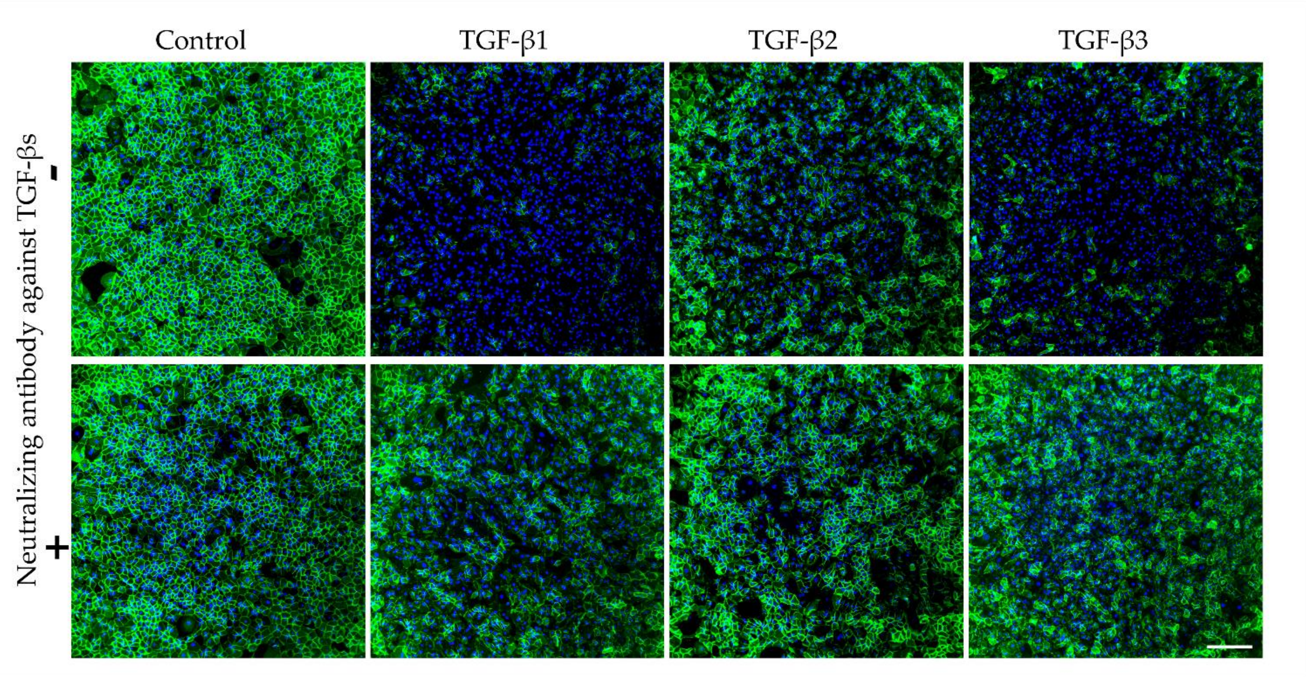

Supplementary Data S2. IF of NCAM on hCECs in SFM-based medium supplemented with or without TGF-β 1, 2, or 3 and in the presence or absence of neutralizing antibody against TGF-βs. Cell lateral membranes were stained in green by IF by NCAM and the nuclei in blue by DAPI. Pictures were obtained using an epifluorescence microscope using the same parameters for all pictures (intensity of light source, exposure time, contrast, resolution). Experiment was repeated twice using two different cell cultures. The objective was X10, scale bar=200 μm.
